# Respiratory Mandibular Movement Signals Reliably Identify Obstructive Hypopnea Events During Sleep

**DOI:** 10.3389/fneur.2019.00828

**Published:** 2019-08-13

**Authors:** Jean-Benoit Martinot, Nhat-Nam Le-Dong, Valerie Cuthbert, Stephane Denison, Jean C. Borel, David Gozal, Jean L. Pépin

**Affiliations:** ^1^Sleep Laboratory, CHU UCL Namur Site Sainte-Elisabeth, Namur, Belgium; ^2^Institute of Experimental and Clinical Research, UCL Bruxelles Woluwe, Brussels, Belgium; ^3^Research and Development Center, Respisom, Erpent, Belgium; ^4^HP2 INSERM U1042, Université Grenoble Alpes, Grenoble, France; ^5^Department of Child Health and Child Health Research Institute, University of Missouri, Columbia, MO, United States

**Keywords:** sleep apnea syndrome, hypopnea, respiratory effort, mandibular movements, obstructive hypopnea, central hypopnea

## Abstract

**Context:** Accurate discrimination between obstructive and central hypopneas requires quantitative assessments of respiratory effort by esophageal pressure (OeP) measurements, which preclude widespread implementation in sleep medicine practice. Mandibular Movement (MM) signals are closely associated with diaphragmatic effort during sleep.

**Objective:** We aimed at reliably detecting obstructive off central hypopneas events using MM statistical characteristics.

**Methods:** A bio-signal learning approach was implemented whereby raw MM fragments corresponding to normal breathing (NPB; *n* = 501), central (*n* = 263), and obstructive hypopneas (*n* = 1861) were collected from 28 consecutive patients (mean age = 54 years, mean AHI = 34.7 n/h) undergoing in-lab polysomnography (PSG) coupled with a MM magnetometer, and OeP recordings. Twenty three input features were extracted from raw data fragments to explore distinctive changes in MM signals. A Random Forest model was built upon those input features to classify the central and obstructive hypopnea events. External validation and interpretive analysis were performed to evaluate the model's performance and the contribution of each feature to the model's output.

**Results:** Obstructive hypopneas were characterized by a longer duration (21.9 vs. 17.8 s, *p* < 10^−6^), more extreme low values (*p* < 10^−6^), a more negative trend reflecting mouth opening amplitude, wider variation, and the asymmetrical distribution of MM amplitude. External validation showed a reliable performance of the MM features-based classification rule (Kappa coefficient = 0.879 and a balanced accuracy of 0.872). The interpretive analysis revealed that event duration, lower percentiles, central tendency, and the trend of MM amplitude were the most important determinants of events.

**Conclusions:** MM signals can be used as surrogate markers of OeP to differentiate obstructive from central hypopneas during sleep.

## Introduction

Hypopnea is the most frequent respiratory event reported during sleep (1). AASM rules recommend, as an option, to sub-divide hypopnea as either obstructive or central, depending on the underlying respiratory effort (RE) (2), reflecting increases, or decreases in the central respiratory command, respectively. The gold standard marker of RE during sleep is the amplitude of the esophageal pressure (OeP) curve, a surrogate of the diaphragmatic muscular contraction in the presence of increased flow resistance within the airways (3). Alternative non-invasive technique for assessing RE such as thoraco-abdominal inductance plethysmography, that detects phase angle differences or inspiratory flow limitation assessed by nasal pressure recordings or snoring loudness remain to be validated against OeP for routine hypopnea characterization (2, 3). Correct characterization of the hypopnea sub-type provides information about its origin and contributes to the therapeutic personalized decision-making process (4).

We have recently shown that analysis of respiratory mandibular movements (MM) during sleep reproducibly and reliably identifies RE in patients being evaluated for suspected obstructive Sleep Apnea Syndrome (OSAS). The amplitude of MM mid-sagittal and vertical displacements at the breathing frequency change across different types of scored events similarly to the amplitudes of the EMG activity of the crural diaphragm (5). These findings strongly suggest that MM amplitudes reflect the intensity of RE (6), and also that hypopneas characterized by different levels of RE reflecting more or less recruitment of the central ventilatory command can be potentially identified and, as such, serve as a reliable marker of OeP, a technique that is seldom, if ever, implemented in clinical sleep studies (5).

In this study, we aimed to identify in patients being evaluated for suspected OSAS, whether hypopneas scored as either obstructive or central, based on the OeP measurement and strictly following 2012 AASM rules, would be predictably identified by MM analyses. To this effect, MMs were recorded as time series data and, in the context of the large amount of raw data acquired, we searched for surrogate features of specific patterns to make quantitative comparisons between the scored hypopneas.

## Materials and Methods

### Study Subjects

Thirty-six consecutive adult patients referred for suspected OSAS in a single sleep center (CHU UCL Namur, Saint Elisabeth site, Namur, Belgium) were invited to participate. All participants had symptoms suggestive of underlying OSAS. The study was approved by the local human ethics committee (IRB 00004890-number B707201523388), and all participants provided a written informed consent.

### Study Design

This was a prospective cross-sectional study performed during a single night PSG.

### Measurements and Data Acquisition

#### Polysomnography

A commercial digital acquisition system (Somnoscreen Plus, Somnomedics, Randersacken, Germany) was used for recording in laboratory PSG. The parameters monitored included EEG (Fz-A+, Cz-A+, Pz-A+), right and left electro-occulogram, submental EMG, tibial EMG, chest and abdominal wall motion by respiratory inductance plethysmography (SleepSense S.L.P.Inc, St. Charles, IL, USA), nasal and oral flows, respectively with a pressure transducer and a thermistor, and O2 saturation by digital oximeter displaying pulse wave form SpO2 (Nonin, Nonin Medical, Plymouth, MN, USA). Following instillation of local anesthetic, a 2.5 mm external diameter soft silicone covered catheter (Gaeltec Ltd, Dunvegan, Isle of Skye, Scotland, UK) was inserted through the nares into the esophagus.

The catheter is mounted with 1 pressure transducer, which is a thin film resistive strain gauge sensors. Proper positioning of the catheter was verified by visual inspection of the signal itself. The catheter was secured with tape to the patient's nose, lip, and cheek. The transducer was calibrated relative to atmospheric pressure (zero) before each recording.

The catheter was connected to a miniature computer recorder (digitraper) that is placed on the bedside locker overnight (7).

#### Mandibular Movements (MM)

MM were assessed with a midsagittal mandibular movement magnetic sensor (Brizzy® Nomics, Liege, Belgium) which measures the distance between two parallel, coupled, resonant circuits placed on the forehead, and on the chin. It was used to record mandibular movements (8). The transmitter generates a pulsed magnetic wave of low energy. The change in the magnetic field recorded at the receiver is inversely related to the cube of the distance between the chin and forehead probes. The distance between the two probes is measured in mm with a resolution of 0.1 mm. Basically, this signal provides the instantaneous position of the mandible (e-Figure 1).

#### Polysomnography Scoring

PSG scoring (sleep stages and respiratory events) was performed by two trained technicians who were blinded to the study aims and an in strict accordance with the American Academy of Sleep Medicine rules (2).

Analysis was restricted to 28 of the 36 originally recruited patients who spent a minimum of 4 h sleeping along with good quality signals on all recorded channels including the OeP. Normal breathing periods (NPB), central, and obstructive hypopnea events were scored.

Scoring of the conventional polysomnography supplemented with Esophageal pressure measurement showed a strong agreement between the two readers: ICC (2.1) = 0.927 (95%CI: 0.901–0.962; *p* < 0.001).

A hypopnea was defined as a reduction in nasal pressure signal (flow) of > 10 s, ended by an arousal or a decrease in SpO2 of at least 3% relative to baseline. Hypopneas were characterized in obstructive vs. central events depending on the presence or absence of RE during at least one respiratory cycle. This was assessed by changes in OeP swings accompanied by at least one other PSG signal reporting RE (namely, flow pressure limitation, respiratory belt asynchrony, or snoring). OeP swings consisted in progressively more negative amplitudes terminated by a sudden increase to a less negative level.

A central hypopnea was identified if there was a clear reduction in OeP swings from baseline along all the episode time (3). The hypopneas combining periods of no RE or decreasing RE and then at least one respiratory cycle with marked increasing RE (mixed hypopneas) were scored as obstructive (2). An example of fragment is shown in e-Figure 1 after unblinding.

Nevertheless, to optimize the validity of labeling, only the labels which represent a perfect agreement between 2 scorers have been included for main analysis. Ambiguous fragments were excluded from training data.

#### Data Processing Analysis

**Figure 1:**
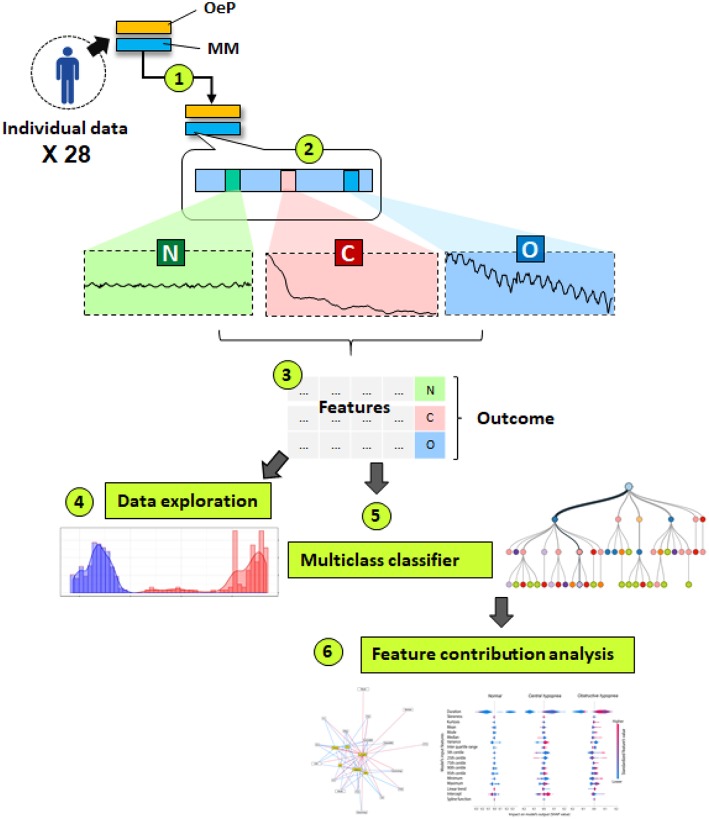
This schematic diagram summarizes a 6 steps data analysis plan. (1) Acquisition and preprocessing of individual data in 28 patients. OeP, Esophageal pressure signal; MM, Mandibular movement signal; (2) Manual label scoring based on OeP signal: N, Normal breathing (*n* = 501), O= Obstructive hypopneas (*n* = 21861) and C, Central hypopneas (*n* = 263); (3) Feature extraction and data compilation; (4) Exploratory data analysis; (5) Developing a classification rule based on Random Forest algorithm; (6) Model explanation, to determine the role of each contributor and their interactions to identify the 3 target events.

The analysis plan is summarized in the Figure 1. Feature extraction, data processing and descriptive were done in R statistical programming language (8), while Machine learning experiments were conducted using sci-kit learn and SHAP packages in Python language.

**Figure 2:**
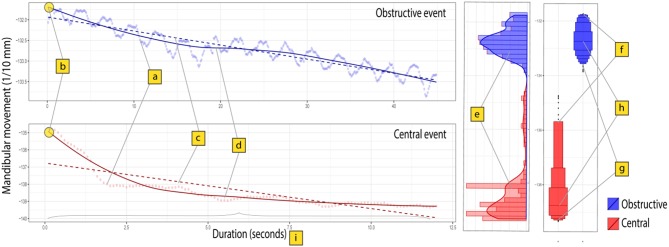
Surrogate features to describe the characteristics of MM signal during central and obstructive hypopnea events. Mandibular movement signal was recorded as a time series (sampling rate = 10 Hz). The figure presents two distinct series: a 12.5 s central hypopnea event (125 points, in red) and an obstructive hypopnea event (45 s or 450 points, in blue). Each point (a) indicates a single value in the series. The slope of a linear model (c) allows to describe the linear trend of MM signal. A generalized additive model (b,d) with polynomial smoothing spline function was fitted for estimating the time dependent variability of MM signal in 2 series, then the intercept (b) and 4 coefficients of spline function (denoted as S1, S2, S3, and S4) were extracted from the model as surrogate features to characterize the complex trajectories of MM in time. The distribution shape parameters (e) (skewness, Kurtosis, Variance) were estimated to describe the shape of MM signal distribution in each series. Other parameters aimed to describe the centrality (h), including mean, mode and median), lower extremities (g, including the minimum, 5th and 25th centiles) and upper extremities (f, including maximum, 95th and 75th centiles) of MM amplitude. Finally, the event duration (i, measured in second) was also included as characteristic feature.

1. After PSG scoring, individual raw data were acquired from 28 patients. Each dataset contained OeP and MM signals (synchronized at 10 Hz frequency and processed with noise reduction). From this database, 2625 fragments including raw MM signal during normal breathing periods (NPB; *n* = 501), obstructive hypopneas (*n* = 1,861), or central hypopneas (*n* = 263) were analyzed.
2. A customized algorithm (e-Table 1) was applied to extract 23 features from MM raw signal of each event (Figure 2) or each 10 s of NPB (9). Those features included: the central tendency (mean, median and mode) of MM amplitudes; MM distribution (raw or enveloped signals): skewness, Kurtosis, IQR, 25th, 75th, and 90th centiles; extreme values: Min, Max, 5th and 95th centiles of MM amplitudes; the tendency of variation: linear trend and coefficients of Tensor product-based spline factors (S1, 2, 3, 4) from a generalized additive model to evaluate MM in function of Time; and the duration of each event.
3. The extracted features and corresponding target labels were integrated to a tabular dataset.
4. Exploratory data visualization, one-way ANOVA and pairwise student-t tests with Bonferroni correction were performed to compare 23 MM features among 3 groups: normal breathing, obstructive and central hypopneas. Significance level was set at highly stringent criteria (*p* = 0.001) (10) for null-hypothesis testing.
5. Model development: The data were randomly split into 2 subsets: a larger set (70%) for model development and a smaller set (30%) for model validation. Because the original training set was unbalanced between central (minority class) and obstructive hypopneas (majority class), a synthetic minority over-sampling technique (SMOTE) on the trainset before model development was applied (11).
  A multiclass classification rule was built to classify the 3 groups using 23 input features. This consisted of a Random Forest algorithm that combined 500 distinct decision trees (each one was constructed on a random subset of 5 features).
6. Model interpretation: The content of the Random Forest model was analyzed in order to evaluate the importance of each feature and the possible coalition that contributed to the classification (potential combinations among them to differentiate obstructive from central hypopnea). To evaluate the contribution of each features to the prediction, the Lundberg's Shapley additive explanation (SHAP) method was adopted (12). The theory of this method is explained in the Online Supplement (13, 14).

## Results

### Characteristics of the Studied Population and Scoring Performance

Thirty-six OSA patients were recruited and 28 had at least 4 h of tracings without artifacts. The characteristics of the group are presented in Table 1.

**Table 1:** Characteristics of the OSA patients (*n* = 28).

**Parameter**	**Median**	**IQR**	**95%CI**
Age (year)	54.0	19.5	29.5 – 77.0
BMI (kg/m^2^)	29.6	11.3	22.2 – 51.8
TST (min)	381.0	93.1	242.8 – 522.5
AHI (n/h)	34.7	32.9	2.8 – 95.8
ArI (n/h)	30.9	23.1	13.9 – 86.1

*BMI, Body mass index; TST, Total sleep time; AHI, Apnea/Hypopnea index; ArI, Cortical Arousal index*.

Scoring of the conventional polysomnography including esophageal pressure measurement showed a strong agreement between the two readers: ICC (2.1) = 0.927 (95%CI: 0.901–0.962; *p* < 0.001).

### Exploratory Analysis of MM Signal Features

**Figure 3:**
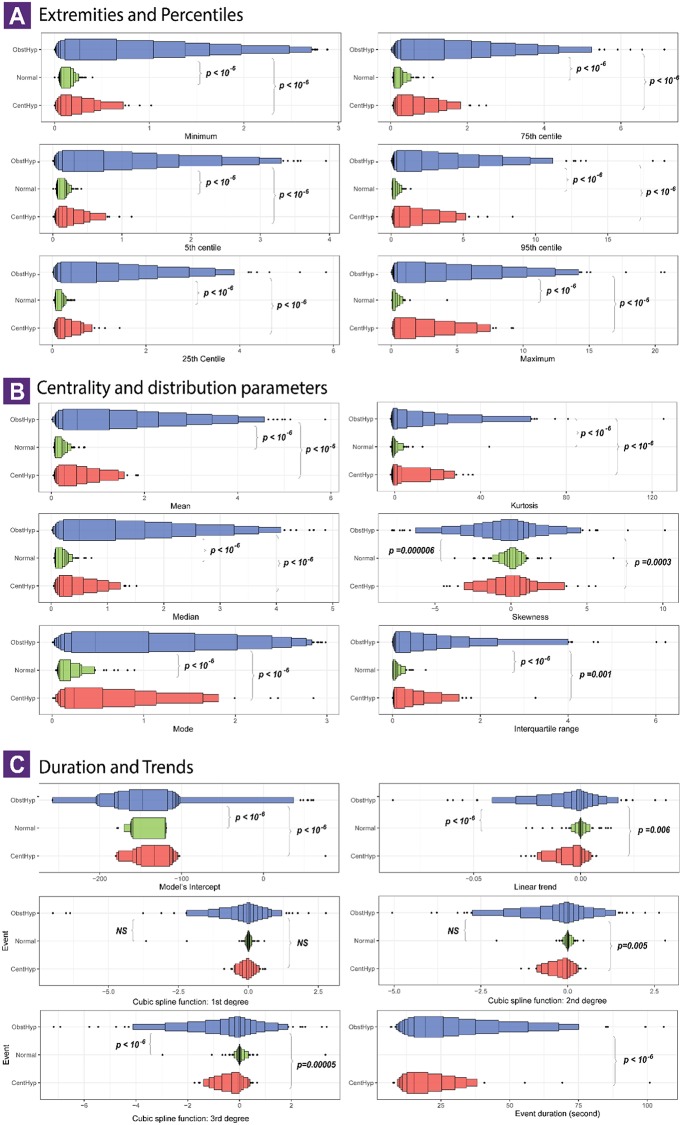
Characteristics of mandibular movement signal during obstructive and central hypopnea events. Each panel visualizes the distribution of a surrogate feature of MM signal in 3 event types: normal breathing (501 series, green color), central hypopneas (*n* = 263 series, red color) and obstructive hypopnea (*n* = 1,861 series, blue color). The surrogate features are presented in 3 groups: **(A)** The extreme levels of signal amplitude, with lower extremities on left side and upper extremities on the right side; **(B)** The centrality or location parameter of the signal (left) and distribution shape parameters (right); **(C)** The event duration, linear trend and coefficients of the smoothing spline time series model (S1–S3). The letter-value boxplot was used to ensure a better description for large data (10). Multiple boxes were drawn, each one represents a pair of lower and upper letter values. The procedure starts with the median, followed with quartiles, and so on. The innermost box is equivalent the conventional boxplot. As moving toward the tails, the boxes became incrementally narrower until we reached the extremes values (outliers, minimum, and maximum). The p_values correspond to a pairwise comparison using *t*-test with Bonferroni correction. A difference is considered significant if p_value is lower than 10^−6^.

MM signal characteristics were evaluated during obstructive hypopneas (*n* = 1,861), in comparison with NPB (*n* = 501) and central hypopneas (*n* = 263). The first features group measured the lower and upper extremities of MM amplitudes. As presented in the Figure 3A, there was a clear contrast between the two types of hypopnea in terms of extremity levels of MM amplitude. All features, including minimum, maximum and 4 centiles (5th, 25th, 75th, and 95th) were 2 to 4 times larger during obstructive hypopnea compared to central hypopnea events (all differences were significant at *p* value thresholds below 10^−6^).

The second feature set describes the central tendency (mean, median, mode), the dispersion (interquartile range) and the distribution shape (skewness and kurtosis) of MM signals (Figure 3B).

NPB were characterized by a steady and symmetrical MM pattern with the amplitude centralized at zero. By contrast, there was a high variability in distribution of MM values during hypopnea events, that could be symmetrical, right or left skewed, leptokurtic, or platykurtic. The differences were consistent for all 3 centrality parameters, suggesting that obstructive hypopneas had a significantly higher MM amplitude compared with central hypopnea events (*p* < 10^−6^). No significant difference was found in terms of skewness and kurtosis.

The third feature set allows to capture the duration (in s), the linear and the curvilinear trends of MM in function of time (Figure 3C). Compared to the central events, the obstructive events had a significantly longer duration (21.9 vs. 17.8 s, *p* < 10^−6^). During NPB, the MM trend was null, confirming that MM signal was in a steady state. The linear and curvilinear trend of MM time series became negative during central and obstructive hypopneas, though no significant differences emerged between these two groups.

### MM Based Classification Rule to Differentiate Central and Obstructive Hypopneas

#### Model's Performance by External Validation

The optimized Random Forest model implied randomly 5 features for each one in 500 different decision trees. When validated on unseen data (*n* = 788 events), the model showed a good performance to classify the 3 classes (normal breathing, central and obstructive hypopneas), with a balanced accuracy of 0.876 and a high agreement with the manual blind scoring based on esophageal signal (Cohen's Kappa coefficient = 0.879). A confusion matrix of the model validation is provided in the Online Supplement (e-Figure 2).

#### Model Interpretation

The interpretation consisted in two steps: (1) Understanding the model structure and (2) Evaluating the contribution of the features to the model's output (e-Figure 3).

**Figure 4:**
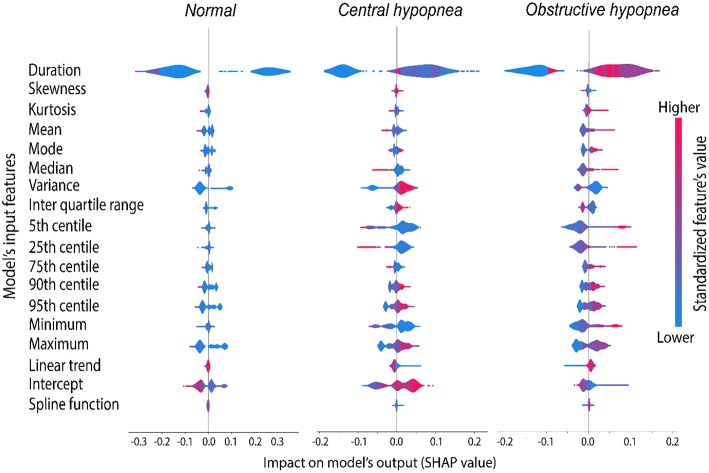
Average marginal contribution of the 18 most relevant features to the model's prediction. Each line in this graph corresponds to a single feature and consists of 3 violin plots showing the contribution of that feature to the prediction of 3 target groups. Each column corresponds to a target group (normal, central and obstructive hypopneas). The violin plots and X scale indicate the distribution of the SHAP value, a score assigned to each feature to measure the average marginal contribution of that feature across all possible coalitions with other features to make a certain prediction. The shape and location of the violin plots indicate the impact of each feature on the model's output, or how much a feature may contribute to a certain prediction. A negative SHAP value indicates that the feature participates to rule out that group (by decreasing the predicted probability), whilst a feature with positive SHAP value would increase the probability of that group, thus supporting its identification. Larger absolute SHAP scores (the density plot extends further to the left or right side) indicate more important role of that feature. A zero SHAP value (concentrated density plot) indicates that the feature does not contribute at all to the prediction of that group. The feature values were normalized and mapped to a blue/red color scale (blue = lower values, red = higher values), allowing to estimate the tendency of prediction as the feature value increases or decreases.

The model's complex structure is described through a network by examining all possible interconnection among 23 features across all decision trees (Figure 4). The network showed that all 23 features contributed to the model's structure, although some features may be more important than others. The event duration played a central role, it was present in all decision rules and collaborated with every other feature. The 5th centile, linear trend and minimum values also had an important role, as these features were linked to many other features in more than 250 rules. The variance of MM amplitude and intercept was also highlighted as these emerged as important elements in the network. The connection among those 6 relevant features formed the core of the ensemble model.

**Figure 5:**
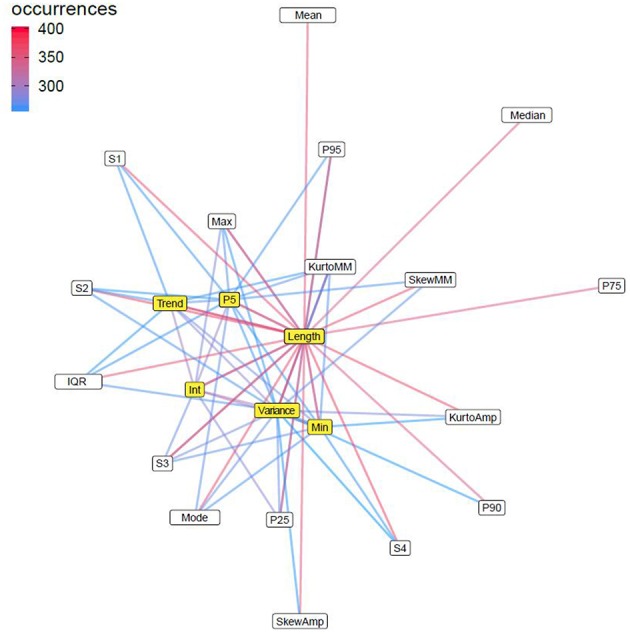
Cooperation network among MM signal features in Random Forest classifier. The figure presents a network of all possible combinations among the 23 potential surrogate features of MM signals that occurred in 500 classification rules in the Random Forest model. Pi, ith percentile; IQR, interquartile range; Amp, amplitude of MM signal; Int, intercept of the linear model estimating MM signal amplitude in function of time; S1–S4, spline functions determining the curvilinear trend of MM signal amplitude in function of Time; Skew, skewness; Kurto, Kurtosis; Trend, linear trend of the MM signal. Each node in the network indicates a feature and the link connecting between two nodes indicates that these two features did co-exist in at least 250 decision trees. The color intensity of connection links is proportionate with the frequency of that combination. The nodes are positioned in function of their ability to inter-connect with other nodes, thus the more centralized nodes (colored in yellow; more links), such as event duration (length), 5th centile, linear trend, intercept, variance, and Min were the most important features, because those features did participate in almost every classification rule.

According to the SHAP value analysis (Figure 5, e-Figure 4), the contribution of the input features could be interpreted as follows:

The most relevant features allowing to distinguish central from obstructive hypopnea included: event duration, variance in signal amplitude, lower extremities (minimum, 5th and 25th centiles), while the central tendency (mean, median and intercept), and the linear trend of the signal showed only moderate contribution.

More specifically, a longer event duration, higher value of centrality (mean, median), and more important linear trend supported the prediction of obstructive hypopnea; in contrast, a shorter event duration, higher values of minimum, 5th and 25th centiles, lower values of mean, or median allowed to rule-out the obstructive hypopnea. On the other hand, a coalition of moderate event duration, lower central tendency (mean, mode or median), lower values of minimum, 5th and 25th centiles, and less important linear trend predicted central hypopnea.

Although the upper extremities (Max, 90th, or 95th centiles), as well as the intercept were found relevant for the prediction, their contributions were equally distributed in both hypopnea types, and thus remained impervious for either central hypopnea or obstructive hypopnea. Other features such as skewness, interquartile range, 75th centile and spline functions were least important as their contribution rarely impacted the model's output (SHAP values were close to zero).

## Discussion

This study expands on our previous findings (5) indicating the clinical utility of MM signals as a surrogate marker of RE during sleep, by extracting more features from the raw MM data and focusing on the differentiation between obstructive and central hypopnea events, an issue that provides more informative content than the general classification of sleep breathing disorders. The Random Forest algorithm was adopted as a statistical inference tool because it offers the capacity to handle multiclass problems as well as delineate complex interactions among input features. The findings not only confirmed the ability of MM to differentiate obstructive from central hypopneas and periods of NPB, but also provided enhanced understanding about the changes in MM signal patterns during these episodes. In general, hypopneas can be reproducibly and correctly characterized as either central (without signs of increasing RE) or obstructive (presence of marked or increasing RE) when considering an ensemble of statistical features in sleep MM signals.

We have previously shown that MM signals provide accurate estimates on the degree of RE in patients with OSAS (15). In a group of consecutive patients clinically referred for evaluation in the sleep laboratory for suspected OSA, MM analyses allowed for successful detection of RE as defined by the conventional scoring rules. Moreover, as shown herein MM can readily differentiate between obstructive and central hypopnea events.

Since many patients exhibit a preponderance of events during sleep that are scored as hypopnea rather than apnea, and since obstructive hypopnea likely share the same pathophysiological determinants and obstructive apnea (4), the AHI does not provide accurate clinical risk stratification since it includes both central and obstructive events, and therefore other PSG-derived features are needed to better define sleep-disordered breathing (16). In the context of the last iteration of AASM scoring rules in 2012, it did not seem that differences with previous scoring guidelines would enhance the prediction of the risk of cardio-vascular morbidities (17). However, the importance of identifying the sub-type of hypopneas to better stratify morbidity risks and overall outcomes cannot be over emphasized.

The current study pointed to both predictive and interpretative goals. However, there is always a trade-off between the predictive power and the interpretability when adopting a statistical learning algorithm. Due to the high dimensionality of the extracted features data and multiclass classification problem, we had to adopt the Random Forest classifier.

We found, however, that both the extremities (min, max) and the lower centiles of the MM amplitudes, as well as the central tendency parameters (mean, median or intercept of a linear regression) provided informative content about the position of the mandible during sleep events, and that obstructive hypopneas lasted longer as compared to central hypopneas. The parameters of the values distribution shape showed no significant differences. In contrast, a smaller variance in the mandibular position and in peak amplitudes was more typically observed during central hypopneas.

Similarly, the linear and the curvilinear trend analyses along the hypopnea spectrum highlighted that the obstructive events generated a more negative signal (mouth more open). The coefficients of the spline function in the non-linear trend analysis were similar between both hypopnea sub-types; it is likely because the signal curve shape of the temporal series was dependent on the individual characteristics. Notably, hypopnea duration contributed importantly to the distinction between central from obstructive hypopnea, the latter being more likely to be of longer duration. Those findings were consistent in both traditional statistical inference (ANOVA) and model interpretative analysis using the SHAP method.

The importance of identifying the sub-type of hypopneas to better stratify morbidity risks and overall outcomes of sleep apnoea patients is now widely recognized. Identification of central breathing events without RE or with decreasing RE is paramount to formulate tailored treatment decisions (18). Indeed, emergence of central respiratory disturbances provides clinically important information on underlying pathological conditions: frequent sleep/wake transitions, excessive loop gain with or without excessive arousability and sleep fragmentation, prolonged circulatory time as in congestive heart failure with increased risk for Cheyne-Stokes breathing, or central alveolar hypoventilation of various causes (19–22). However, all these conditions associated with central hypopnea tend in general to result in shorter events. Episodes, including those with longer decreases in RE are in fact of mixed nature, and were regrouped in this study with the obstructive hypopneas. Previous algorithmic analyses had to consider multiple PSG parameters in combination (flattening of nasal flow-pressure signal, paradoxical breathing, arousal, event termination profile, and sleep stage) to characterize the hypopnea with a satisfactory overall accuracy. In the search for a useful single signal source that manifest overnight stability in the absence of magnetic interferences, MM emerges as a good candidate that can be used in machine-based classifiers aiming at separating central from obstructive hypopneas.

## Limitations

Similar to any study, our study has a few important limitations that deserve comment. Even though a classification rule was built in our analyses, this approach was simply aimed as an experimental demonstration of feasibility and proof-of-concept, rather than reflecting a final and authoritative diagnostic rule. Extracted feature-based algorithms may not be the best solution for clinical practice, due to their higher computation cost and other biases. Therefore, more advanced methods such as recurrent or convolutional neural networks are more efficient for bio-signal learning, pattern recognition, as these algorithms can innately handle both feature extraction and model optimisation, independently from human knowledge-based interventions. As such, implementation of the approaches proposed herein should incorporate a multicentre large cohort from which derivation of more robust and validated rules could be then implemented and disseminated.

We should also point out the imbalance among the 3 event groups, as central hypopneas were less frequent compared to the larger number of obstructive events. However, this problem was handled by conducting an over-sampling process on the training set before building the model and by setting a high threshold of statistical significance for null hypothesis testing (*p* < 10^−6^).

## Conclusions

The signal characteristics of MM can be used as surrogate markers of OeP to correctly classify obstructive and central hypopneas as well as periods of NPB in patients being evaluated for suspected OSAS. MM signal opens doors to automate the more complex part of respiratory events scoring. It also opens the possibility of scoring by less trained physicians and reduces medical errors.

## Data Availability

The datasets analyzed in this manuscript are not publicly available. Requests to access the datasets should be directed to martinot.j@respisom.be.

## Author Contributions

J-BM, JB, DG, and JP designed research. J-BM, VC, SD, and JB performed data acquisition and interpretation. N-NL-D analyzed data. VC and J-BM performed *post-hoc* validation and interpretation of the findings. N-NL-D, DG, J-BM, and JP wrote the paper. JB, DG, and JP revised the manuscript. J-BM is the guarantor of the paper. The final manuscript has been reviewed and approved by all authors.

### Conflict of Interest Statement

The authors declare that the research was conducted in the absence of any commercial or financial relationships that could be construed as a potential conflict of interest.

## Abbreviations

AASM: American Academy of Sleep Medicine
EMG: Electromyography
MM: Mandibular movement
NPB: Normal Period of Breathing
OeP: Esophageal Pressure
OSAS: Obstructive Sleep Apnea Syndrome
PSG: Polysomnography
RE: Sleep Respiratory Effort
SpO2: Pulsed O2 Saturation.
