# Supplementary material for: Respiratory Mandibular Movement Signals Reliably Identify Obstructive Hypopnea Events During Sleep
Source: Front Neurol. 2019 Aug 13;10:828. doi: 10.3389/fneur.2019.00828 (PMC6701450; doi:10.3389/fneur.2019.00828)

SUPPLEMENTAL DOCUMENT

e-Figure 1: A 3 minutes fragment of obstructive hypopnea

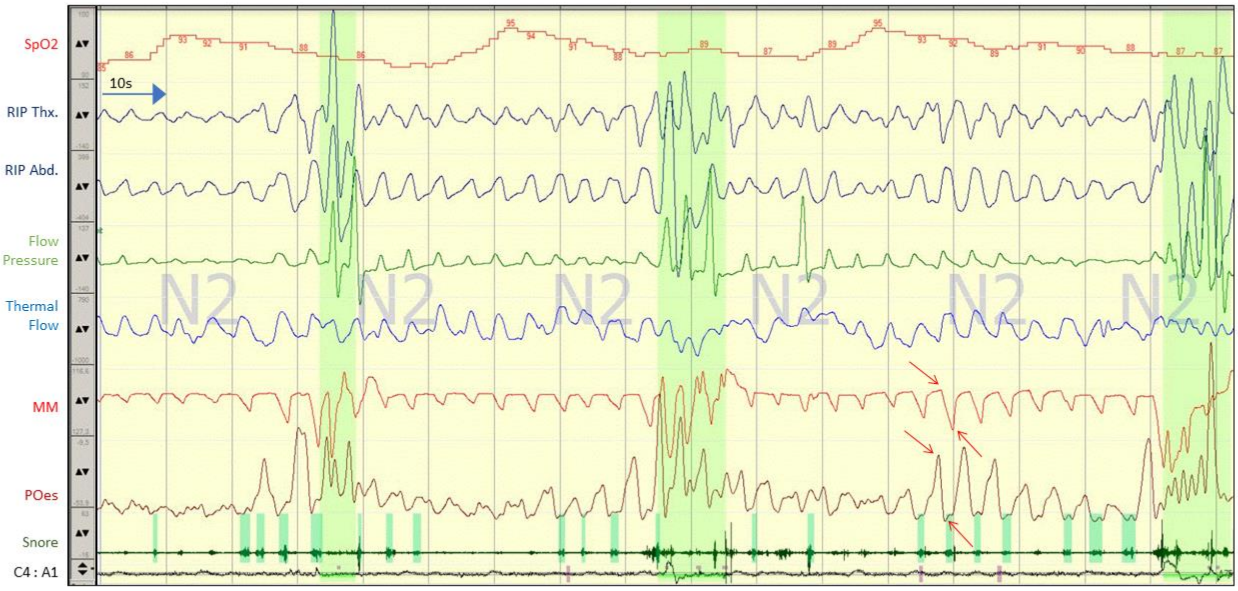

Caption: SpO2: pulsed O2 Saturation, RIP Abd, RIP Thx: Abdominal and thoracic RIP belts respectively, P. Flow: Nasal airflow pressure, Th. Flow: oro-nasal thermal flow, MM: Mandibular Movement, POES: Oesophageal pressure. The red arrows show the values of the peak to peak amplitudes of the mandibular and oesophageal signals, in mm and mm Hg, respectively. The blue arrow shows the time duration between two vertical lines.

e-Table 1

	Feature extraction algorithm
**1**	Loading a long sequence of raw MM data at (sampling rate = 10 Hz)
**2**	Marking the timestamps of Obstructive and Central hypopnea events
**3**	for each time stamps ti, do
**4**	**Check** whether ti is the beginning of an Obstructive or Central hypopnea event?
**5**	If so, assign ti to ***(t_begin),***
**6**	then search for the ending ***(t_end)***
**7**	Extract the raw data sequence to a temporary holder named “**Event E**”, by indexing t_begin and t_end
**8**	**for** each event **E**, **do**
**9**	Calculate event duration ***dt = (t_end – t_begin)***
**10**	Determine the parameters of distribution:
**…**	Min, Max, Mean, median, mode, 5th, 25th, 75th, 90th, 95th centiles, Skewness, Kurtosis, IQR
**11**	Fit a GAM non-linear model to estimate MM amplitude by a spline function on time t,<br>Then extract the coefficient of spline function
**12**	Fit a simple linear model, extract the Intercept and linear slope
**13**	Concatenate all features + labelling
**14**	Pass on next event
**15**	When the last event reached: exit loop

e-figure 2: Confusion matrix showing the model's prediction on validation subset

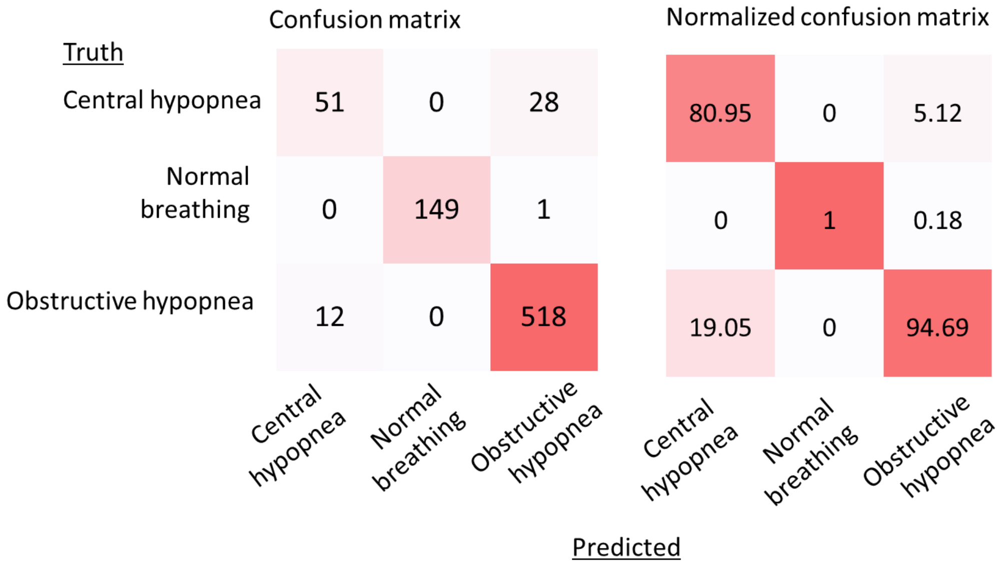

e-figure 3 : Contribution of the most important features to the differentiation of 3 target labels

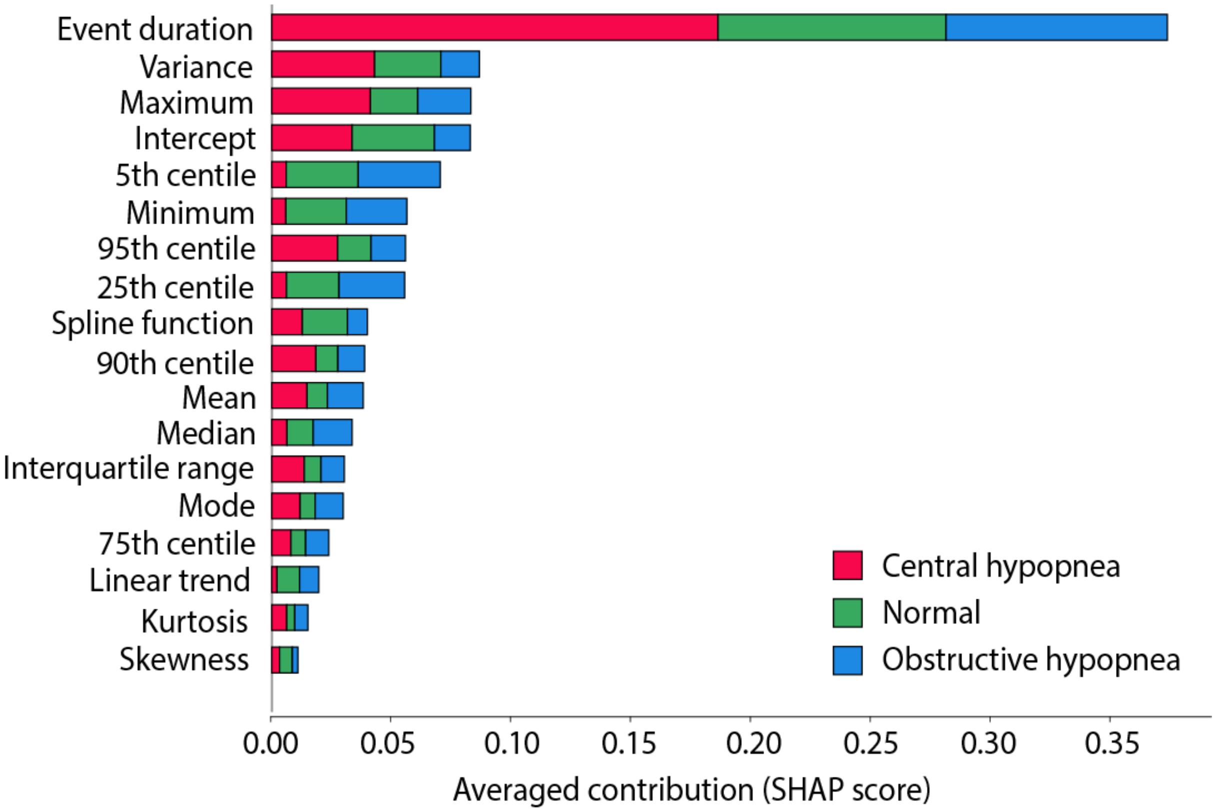

Caption: This graph ranks the 18 most important MM signal features by their global impact on the model's prediction. The bars indicate the mean SHAP score for each feature, stratified by 3 target labels Central hypopneas (Red), Normal (Green) and Obstructive hypopneas (Blue). The SHAP score measures the average marginal contribution across all possible coalitions with other features to classify 3 target labels. The higher SHAP score, the more important contribution that feature may provide.

Note:

The Lundberg's Shapley additive explanation (SHAP) method unified the Shapley's score in cooperative game theory (1953) (13) and the local interpretation approach (14) to provide the best solution so far to explain any black-box model. The SHAP theory considers the input features as "players" in a cooperative game were the "payout" is making correct prediction of a target label (i.e central or obstructive hypopnea). The SHAP algorithm lets each feature value to join with other features in random order to form a coalition, then assign a payout (SHAP score) for each feature values depending on their contribution to the total prediction. The

SHAP score is the result from averaging the change in prediction that a coalition gains when a new feature participated. In essence, SHAP score of a feature value is the average marginal contribution of that feature value across all possible coalitions for a particular prediction.

e-figure 4: Model explanation applied to individual predictions

a) Instruction to read an individual explanation graph

Individual interpretation based on SHAP score

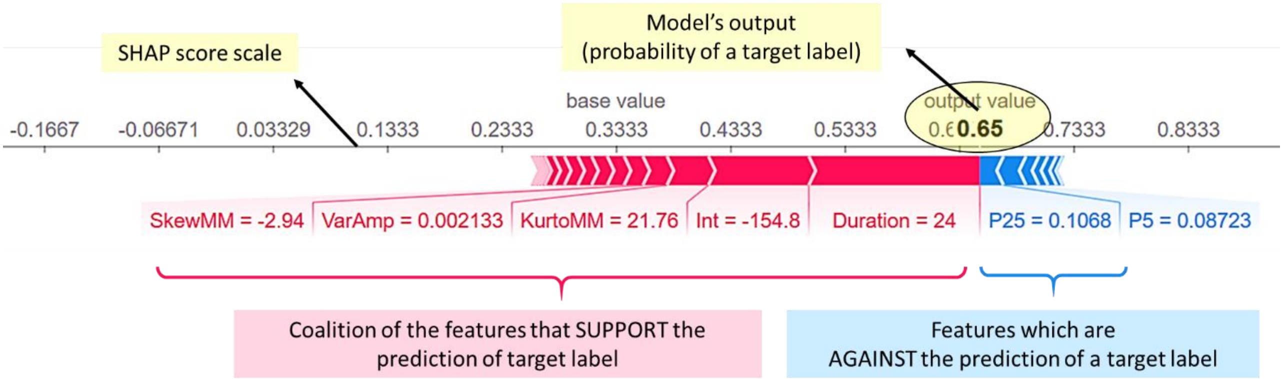

b) Central hypopnea events:

(b1)

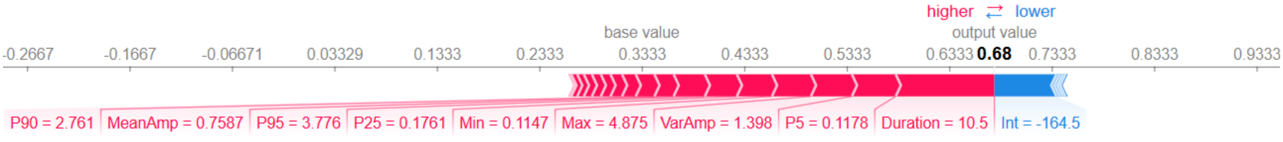

(b2)

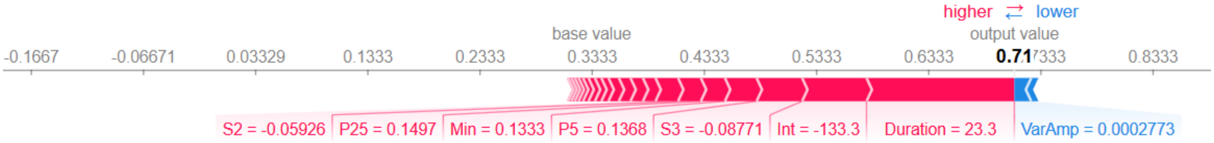

c) Obstructive hypopnea

(c1)

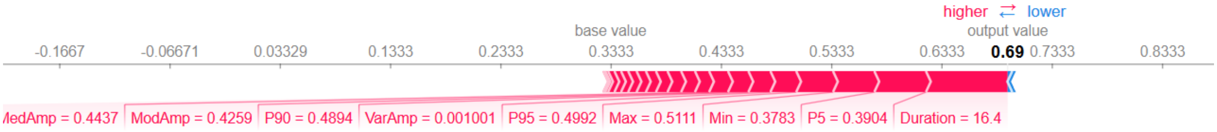

(c2)

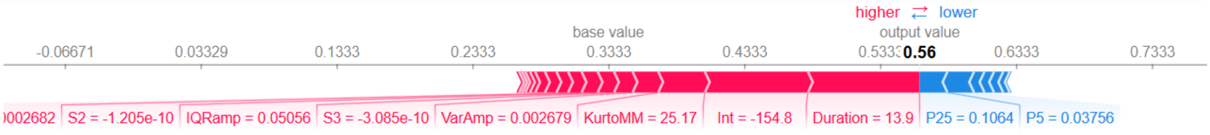

Supplement: Supplementary file 1 [file Data_Sheet_1.PDF]
